# Photosynthetically produced sucrose by immobilized *Synechocystis* sp. PCC 6803 drives biotransformation in *E. coli*

**DOI:** 10.1186/s13068-022-02248-1

**Published:** 2022-12-27

**Authors:** Gábor Szilveszter Tóth, Vilja Siitonen, Lauri Nikkanen, Lucija Sovic, Pauli Kallio, Robert Kourist, Sergey Kosourov, Yagut Allahverdiyeva

**Affiliations:** 1 Molecular Plant Biology, Department of Life Technologies, University of Turku, 20014 Turku, Finland; 2 Cell and Protein Engineering, Institute of Molecular Biotechnology, Graz University of Technology, 8010 Graz, Austria

**Keywords:** Cyanobacteria, Sucrose production, Biotransformation, *E. coli*, Alginate, Immobilization, Cyclohexanone monooxygenase, *ε*-Caprolactone

## Abstract

**Background:**

Whole-cell biotransformation is a promising emerging technology for the production of chemicals. When using heterotrophic organisms such as *E. coli* and yeast as biocatalysts, the dependence on organic carbon source impairs the sustainability and economic viability of the process. As a promising alternative, photosynthetic cyanobacteria with low nutrient requirements and versatile metabolism, could offer a sustainable platform for the heterologous production of organic compounds directly from sunlight and CO_2_. This strategy has been applied for the photoautotrophic production of sucrose by a genetically engineered cyanobacterium, *Synechocystis* sp. PCC 6803 strain S02. As the key concept in the current work, this can be further used to generate organic carbon compounds for different heterotrophic applications, including for the whole-cell biotransformation by yeast and bacteria.

**Results:**

Entrapment of *Synechocystis* S02 cells in Ca^2+^-cross-linked alginate hydrogel beads improves the specific sucrose productivity by 86% compared to suspension cultures during 7 days of cultivation under salt stress. The process was further prolonged by periodically changing the medium in the vials for up to 17 days of efficient production, giving the final sucrose yield slightly above 3000 mg l^−1^. We successfully demonstrated that the medium enriched with photosynthetically produced sucrose by immobilized *Synechocystis* S02 cells supports the biotransformation of cyclohexanone to *ε*-caprolactone by the *E. coli* WΔ*cscR* Inv:Parvi strain engineered to (*i*) utilize low concentrations of sucrose and (*ii*) perform biotransformation of cyclohexanone to *ε*-caprolactone.

**Conclusion:**

We conclude that cell entrapment in Ca^2+^-alginate beads is an effective method to prolong sucrose production by the engineered cyanobacteria, while allowing efficient separation of the cells from the medium. This advantage opens up novel possibilities to create advanced autotroph–heterotroph coupled cultivation systems for solar-driven production of chemicals via biotransformation, as demonstrated in this work by utilizing the photosynthetically produced sucrose to drive the conversion of cyclohexanone to *ε*-caprolactone by engineered *E. coli*.

## Background

Microbial cell factories operating under mild, physiological conditions offer a sustainable production platform for replacing fossil-based fuels, and greener production of high value chemicals like vitamins, flavonoids as well as compounds used in polymer industry like polylactic acid, polyhydroxybutyrate and *ε*-caprolactone [1–8]. One of the promising cell factory concepts is whole-cell biotransformation, where cells transform externally supplied substrate(s) into a product(s). This approach can be used to catalyze several different types of reactions like in our case converting ketones to lactones by a Baeyer–Villiger monooxygenase (BVMO) [7]. *ε*-caprolactone is produced in an industrial scale as a precursor for polycaprolactone and nylon. The industrial process involves the use of peracids as oxidative agents, which are produced in an environmentally harmful way [9]. The enzymatic process harbored in microbial cells use molecular oxygen thus reducing the environmental cost of the process [7]. The advantages of whole-cell biotransformation over the use of industrial chemical catalysts are high selectivity, reduced toxic byproduct formation, and the possibility to operate under physiological conditions. However, heterotrophic bacteria and yeast require an organic carbon source to drive the biotransformation process, which strongly decreases the sustainability aspect of the production system [10, 11]. To circumvent this limitation, photosynthetic microorganisms such as cyanobacteria can be used to drive the process to produce the necessary organic carbon source from atmospheric CO_2_ using light energy. Cyanobacteria can harbor various exogenous synthesis pathways that achieve similar production rates as *Escherichia coli* [12]. Despite the advances in synthetic biology applications, there are still challenges in engineering cyanobacteria for large-scale industrial use [13]. These include the complexity of the photoautotrophic cell where the distribution of photosynthetic energy and CO_2_ are strictly regulated, which can interfere with the bioproduction of specific target chemicals [14, 15]. BVMOs have been successfully expressed in *Synechocystis* sp. PCC 6803 (hereafter *Synechocystis*) but some side-product formation was still detected [9].

In this work, we propose a coupled microbial production system in which the engineered cyanobacterium, *Synechocystis*, functions as a primary source of sugar for driving heterotrophic biotransformation in *E. coli*. Many cyanobacteria are capable of synthesizing sucrose as a compatible solute through an inherent sucrose pathway that natively functions to protect the cell against high extracellular levels of NaCl [1, 16]. In the wild-type strains the synthesized sucrose is retained inside the cells and is therefore not readily available in the culture medium. To enable the coupled production, heterologous sucrose permease CscB (encoded by *cscB* from *E. coli*) has been introduced to various cyanobacteria including *Synechococcus elongatus* sp. PCC 7942, UTEX 2973 and *Synechocystis* [14, 17, 18] to facilitate sucrose transfer into the medium. Using engineered *Synechococcus* strains, this strategy has been demonstrated to sustain heterotrophic growth of microbes such as *Bacillus subtilis, E. coli, Pseudomonas putida, Halomonas boliviensis, Azotobacter vinelandii* and *Saccharomyces cerevisiae*, and used in the production of *α*-amylase, lipids, isoprene and polyhydroxyalkanoates [8, 13, 17, 19–22]. However, to our best knowledge, this is the first study using a *Synechocystis* strain to provide sucrose for a heterotroph to drive a whole-cell biotransformation process.

**Fig. 1:**
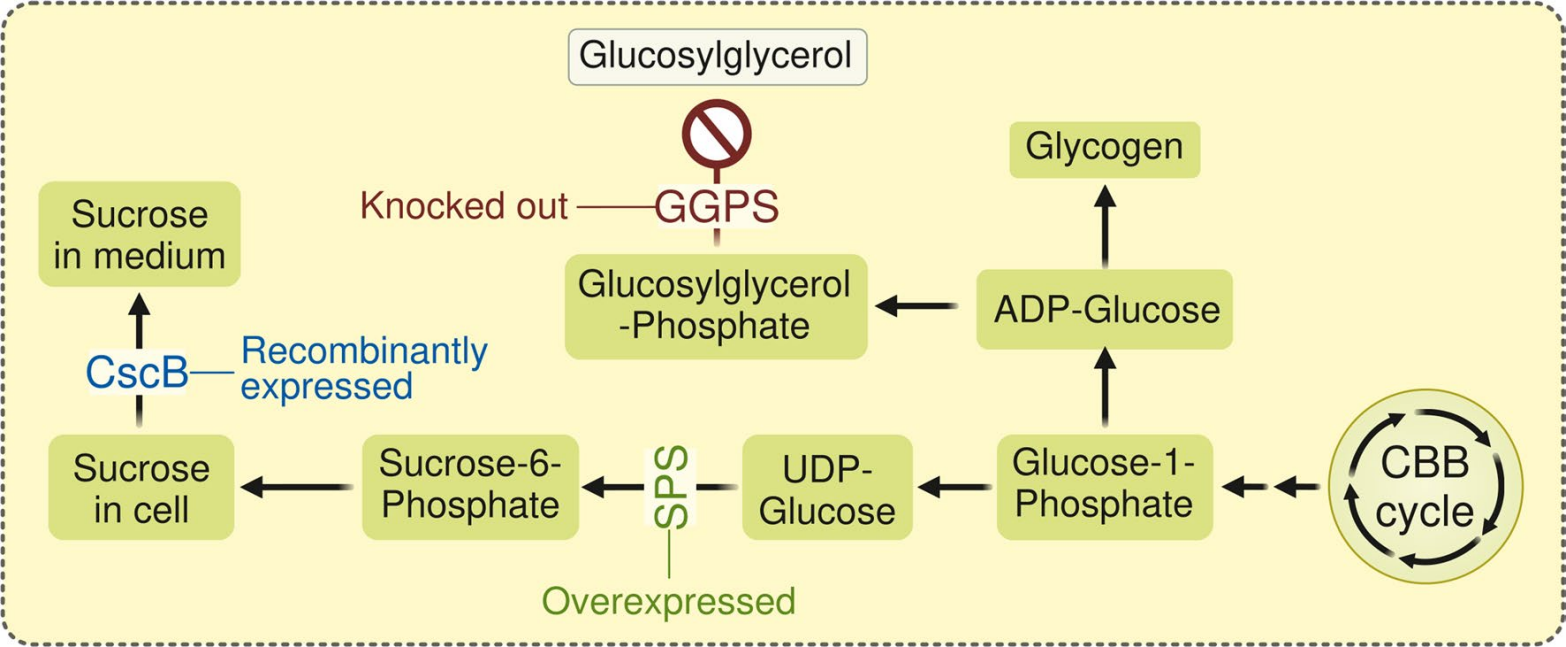
Genetic modifications in sucrose secreting *Synechocystis* S02 strain (adapted from Thiel et al. [14])

Additional genetic modifications have been shown to increase the amount of secreted sucrose, including the inactivation of glucosylglycerolphosphate synthase (GGPS) and overexpression of sucrose phosphate synthase (SPS) in the model strain *Synechocystis*. GGPS is responsible for the synthesis of glucosylglycerol (GG), which alongside sucrose alleviates osmotic pressure in the cells, while SPS is a key enzyme in the sucrose biosynthesis pathway (Fig. 1). The resulting strain S02 (hereafter *Synechocystis* S02) showed the capacity to secrete up to 1800 mg l^−1^ sucrose in suspension cultures under high light conditions (200 μmol photons m^−2^ s^−1^) over 12 days [14].

The concept of this study was to further improve the sucrose productivity by immobilizing the cyanobacterial cells in Ca^2+^-alginate beads. Entrapping the cells in the polymer matrix effectively limits cell division, thereby allowing available resources to be redirected to produce target chemicals instead of promoting growth/biomass accumulation. Immobilization alters the cell metabolism, prolongs production time as well as improves volumetric productivity [23, 24]. Moreover, by separating the cells from medium, immobilized systems save energy on harvesting [25].

**Fig. 2:**
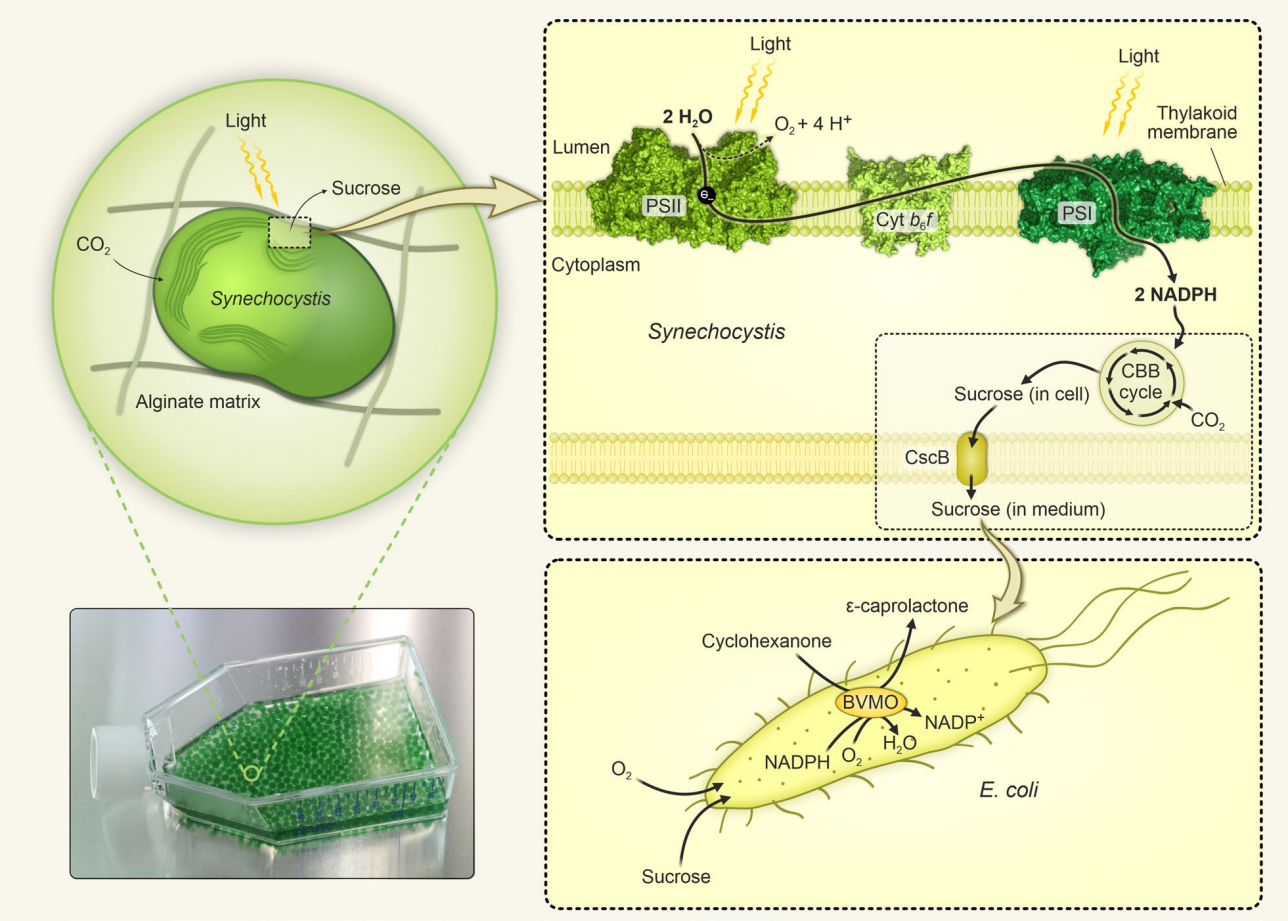
Schematic representation of a coupled production system applied in this work. Photoautotrophically produced sucrose is secreted into the medium by *Synechocystis* S02 cells immobilized in alginate beads. The medium enriched with sucrose is used to drive biotransformation of cyclohexanone to *ε*-caprolactone by an engineered *E. coli*

In the present work, we demonstrate a proof of principle for coupling sucrose production by immobilized *Synechocystis* S02 in batch (where the medium is added in the beginning of the production phase and only gases exchange until termination of the experiment) and semi-continuous (where the medium with the product is periodically replaced with fresh medium, but the immobilized cells are not removed until the end of the experiment) cultivation systems with a biotransformation reaction driven by engineered *E*. *coli*. For this purpose, the medium enriched with sucrose by alginate immobilized *Synechocystis* S02 cells was successfully used for the biotransformation in the recombinant *E. coli* WΔ*cscR* Inv:Parvi. This strain expresses Baeyer–Villiger monooxygenase enzyme from *Parvibaculum lavamentivorans* (BVMO_*Parvi*_) to convert cyclohexanone into *ε*-caprolactone. Furthermore, the strain is able to effectively utilize low amounts of sucrose due to the inactivation of the sucrose catabolism repressor gene *cscR* and heterologous expression of an invertase (Fig. 2)*.*

## Results and discussion

### Specific production of sucrose increases in immobilized *Synechocystis* S02

In order to enhance sucrose production by the engineered *Synechocystis* S02 cells and induce its export to the medium, cultures were resuspended in BG11 + NaCl medium (OD_750_ = 0.5) supplemented with 1 mM IPTG (see the Materials and methods section for more details).

**Fig. 3:**
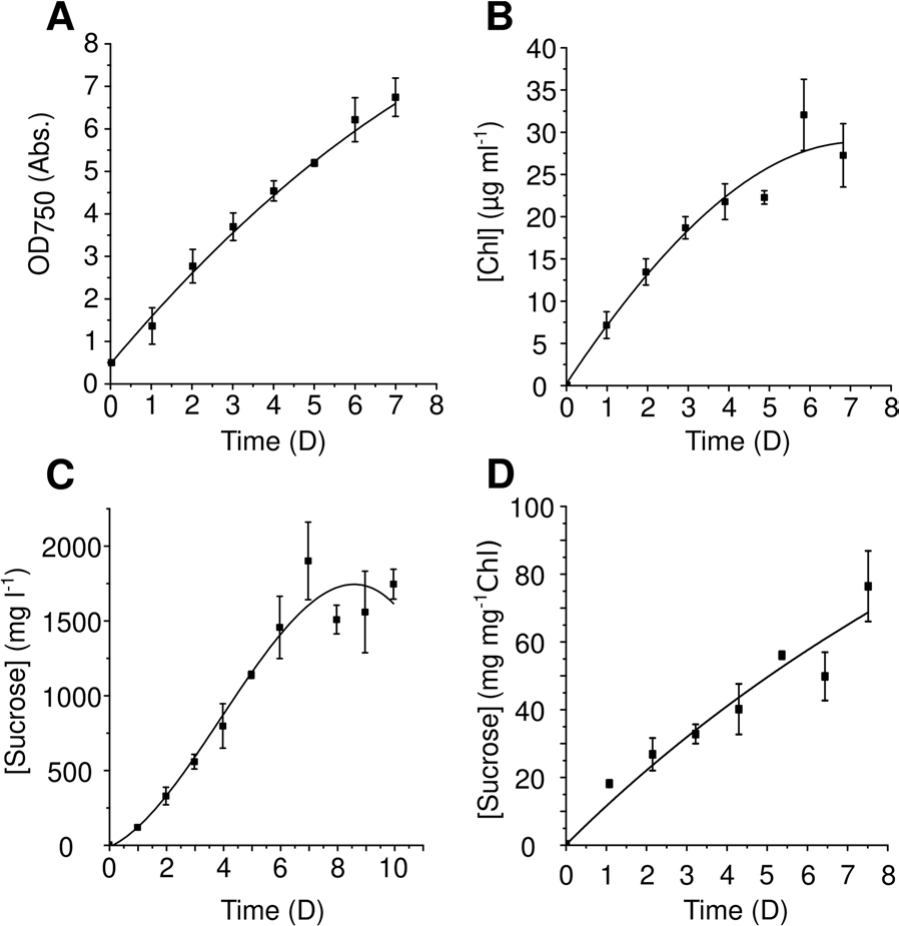
Characterization of sucrose-producing *Synechocystis* S02 in suspension cultures. **A** Cell growth assessed by OD_750_; **B** Chl content; **C** total sucrose concentration in the culture medium; **D** specific sucrose production yield [mg (mg Chl)^−1^]. Error bars represent the standard deviation of three independent biological and two technical replicates

Suspension cultures of sucrose-producing *Synechocystis* S02 showed almost linear growth during 7 days of incubation reaching optical density at 750 nm (OD_750_) of ~ 6.8 (Fig. 3A). Simultaneously, chlorophyll *a* (Chl) concentration steadily increased and saturated at the 6th day (Fig. 3B). Sucrose accumulated in the medium in a linear manner until the 7th day, after which it plateaued at the maximum concentration of 1910 mg l.^−1^ (Fig. 3C), which corresponds to ~ 70 mg sucrose per mg of Chl (Fig. 3D). These results are comparable to the data obtained by Thiel et al. [14]

The entrapment of algae and cyanobacteria in the polymeric matrix is known to diminish cell division and formation of biomass, thus allowing to divert energy for production of targeted chemicals [23, 25]. Such solid-state photosynthetic production systems transfer cells to long-lived biocatalytic production mode. Therefore, in the next step we immobilized the sucrose-producing cells in 3% (wt/v) alginate beads cross-linked with Ca^2+^-ions. The beads can endure vigorous shaking, which facilitates the mass transfer between the cells and the medium through the matrix.

**Fig. 4:**
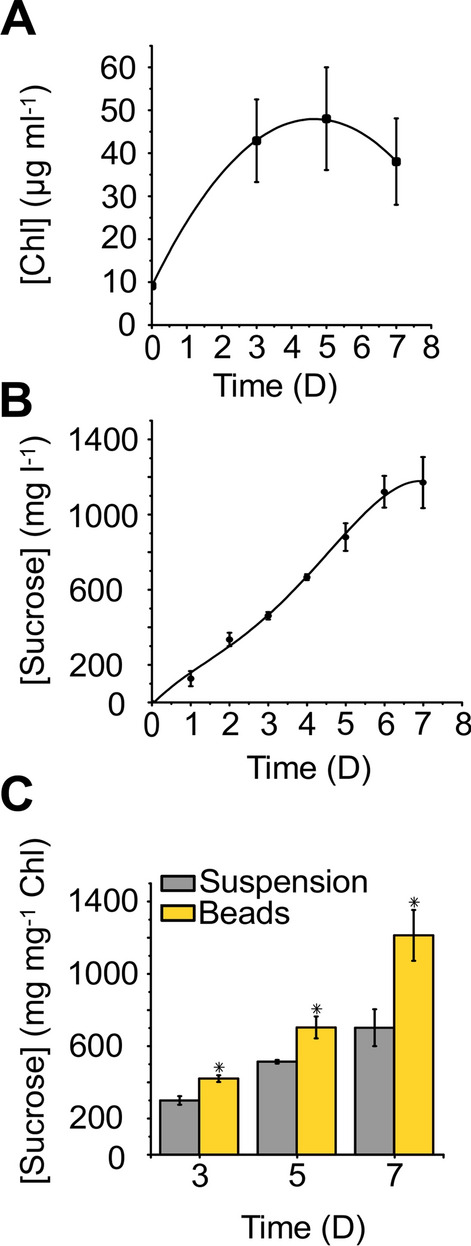
Characterization of alginate immobilized *Synechocystis* S02 cells. **A** Chl content of immobilized cells; **B** sucrose production in alginate immobilized *Synechocystis* S02 cultures; **C** specific sucrose production yields in suspension cultures and immobilized cells. Error bars represent the standard deviation of three independent biological and two technical replicates. The difference between suspension and alginate-entrapped samples is statistically significant for each time point (*P* = 0.001–0.006)

Different from suspension cultures, the immobilized cells showed the most pronounced Chl accumulation during the initial phase of the sucrose production (Fig. 4A).

By the 3rd day of the experiment, the color of the beads became much darker than in the beginning due to the Chl accumulation. Then, the Chl content of the beads increased slower until the 5th day and started gradually declining after that (Fig. 4A). The initial rise in the Chl content can be explained by the cell division and the increase of the biomass within the matrix until the point when the immobilized cultures start experiencing light and presumably nutrient limitations, resulting in inhibition of metabolic activity and cell growth by the end of the experiment. It is important to note that cell outgrowth from the beads was hardly noticeable during 7 days of sucrose production.

Ca^2+^-alginate-entrapped cells produced sucrose similar to suspension cultures (Figs. 4 and 3). The specific productivity of both cultures steadily increased from the beginning of the experiment and reached the maximum on the 7th day, after which production ceased under both setups (Figs. 3C and 4B). It is noteworthy that the immobilized cells showed significantly higher specific production yields compared to suspension cells throughout the experiment reaching the 86% increase (1200 and 700 mg sucrose mg^−1^ Chl, respectively) by the 7th day (Fig. 4C). The total maximum sucrose yield of immobilized cells was 1150 mg l^−1^ (Fig. 4B).

It was demonstrated in other works that the immobilization of *Synechocystis* is an effective way to increase the production yield of the cells. Immobilization in Ca^2+^-alginate beads was reported to increase succinate [26] and *β*-phellandrene [27] production, while immobilization in Ca^2+^-alginate thin film was shown to be effective for the increase of ethylene production [28]. The immobilization was previously reported to be effective to increase sucrose production as well, with engineered *Synechococcus elongatus* sp. PCC 7942 cells showing a 2- to 3-fold increase in specific sucrose production after their entrapment within Ba^2+^-alginate beads [8]. Our results obtained with *Synechocystis* S02 cells immobilized within Ca^2+^-alginate beads are in line with the above-mentioned studies. The production of sucrose by *Synechocystis* cells could be further enhanced by genetic engineering towards constitutive sucrose synthesis without application of the high salt stress and by redesigning the photosynthetic electron transport for enhanced carbon partitioning towards the sucrose production [14, 18]. Further technological improvements are also possible, such as the design and use of specialized photobioreactors with the improved light distribution and the application of new immobilization materials with better porosity and mechanical stability.

### Sucrose production stimulates photosynthetic O_2_ evolution and CO_2_ fixation

**Fig. 5:**
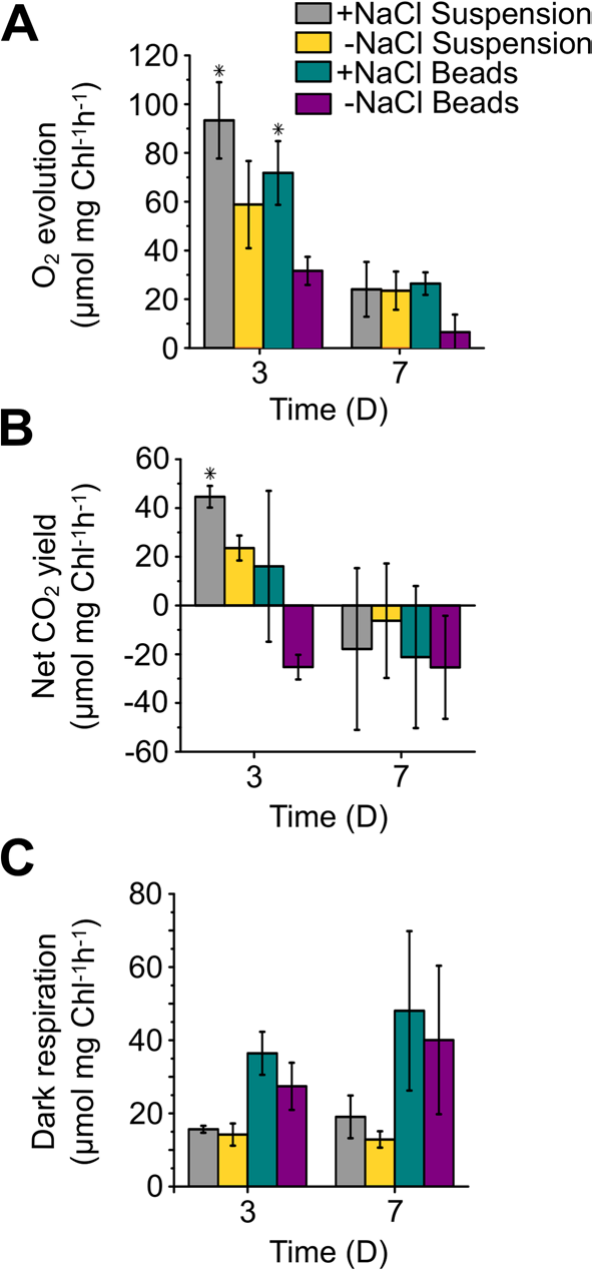
Real-time gas exchange rates in suspension and immobilized cells. **A** Average steady state net O_2_ evolution; **B** net CO_2_ yield; **C** dark O_2_ consumption. The cells were incubated in BG11 supplemented with NaCl and 1 mM IPTG (+ NaCl) to induce sucrose production. As a negative control the cells were incubated in BG11 medium supplemented with 1 mM IPTG, but not NaCl (-NaCl). Error bars represent the standard deviation of three independent biological replicates. Statistical significance between cultures in medium with and without NaCl is marked by * (*P* = 0.006–0.04)

The real-time gas fluxes were monitored in immobilized cells and in cells grown in suspension by membrane inlet mass spectrometry (MIMS) to investigate the correlation between sucrose production and photosynthetic activity. After 3 days of sucrose production, net photosynthetic O_2_ evolution both in suspension and immobilized cultures was significantly higher (*P* = 0.02–0.04) in cultures producing sucrose (+ NaCl) compared to non-producing ones (-NaCl) (Fig. 5A). Net CO_2_ yield rates were also higher in the sucrose-producing cells, albeit the difference was statistically significant only in suspension cultures (*P* = 0.006) (Fig. 5B). These results suggest that sucrose production acts as a strong sink for photosynthetic CO_2_ fixation, which increases the overall photosynthetic activity of the cells. The increase in net O_2_ evolution and net CO_2_ fixation yield in suspension cultures of sucrose-exporting cells was also described previously [14, 29]. It is important to note that we observed higher gas-flux rates in suspension cultures compared to bead-immobilized cells, which can be attributed to the low gas permeability of the alginate matrix and other effects of immobilization on the cell metabolism [25]. By the 7th day, the photosynthetic O_2_ evolution and carbon fixation rates dropped both in the suspension cultures and immobilized cells, demonstrating a decrease in photosynthetic activity. In line with the real-time gas exchange results the effective photosynthetic yield *Y(II)* in both cultures dropped from 0.37 to 0.08, which was accompanied by the decline of sucrose production. A possible reason for the decline in photosynthetic activity is the accumulation of sucrose, which switches the cell metabolism into photomixotrophy, when the cells start metabolizing sucrose as a carbon source while performing photosynthesis and CO_2_ fixation [30]. This form of metabolism, which provides phototrophic cells with extra energy and carbon, often occurs when organic carbon sources are available in the environment, for example during phytoplankton blooms. Besides decreased photosynthetic activity, the transition to photomixotrophic growth is accompanied by increased cell respiration [31]. Indeed, we observed a tendency to enhanced respiration by the end of the experiment almost under all conditions, except in unstressed suspension cultures (-NaCl Susp) where respiration did not change (Fig. 5C). However, based on this data we could not clearly state if enhanced respiration is linked to the transition to mixotrophic growth. Net CO_2_ yield, which represents a difference between CO_2_ consumption in the CBB cycle and CO_2_ release in respiration, was low after 3 days in all cases and dropped below the respiration compensation point by the 7th day. The latter indicates on a significant drop in CO_2_ fixation capacity and correlates well with high respiration rates (Fig. 5C), which finally affect the sucrose productivity. The further studies are needed for understanding mechanisms leading to the transition to the photomixotrophic growth and the declined sucrose production capacity. In the long-term production process, photomixotrophy can be abolished by periodically refreshing the medium throughout.

The cultivation, thus preventing nutrient limitation and sucrose accumulation in the cultures leading to the end-product inhibition effect and further metabolization of sucrose by the cells.

### Semi-continuous production mode leads to prolongation of the sucrose production in beads

**Fig. 6:**
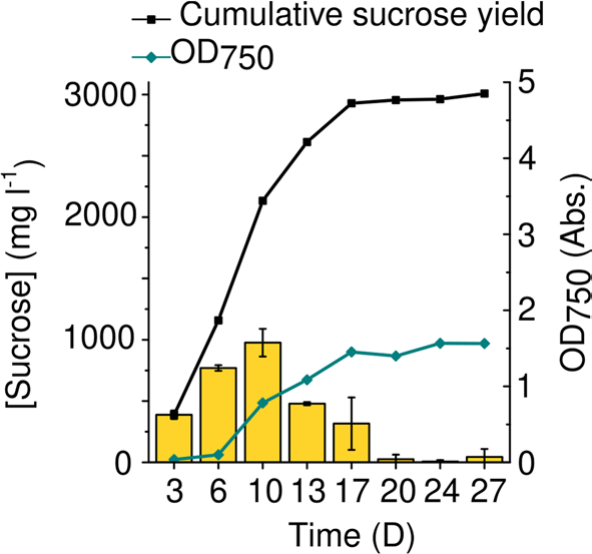
The cumulative amount of sucrose harvested from bead-immobilized cultures. Samples were taken and the medium was refreshed every 3–4 days. Error bars represent the standard deviations of two independent biological and two technical replicates. Cumulative values are obtained by summing up the averages obtained from individual time points. Outgrowth was evaluated by measuring OD_750_ from the medium. OD_750_ data points represent individual measurements

To remove secreted sucrose, the medium was refreshed every 3–4 days after sampling throughout the experiment. By employing this method, the period of efficient sucrose production was considerably prolonged. At day 10, the cumulative production yield was close to 2200 mg sucrose l^−1^ (Fig. 6, black squares) and then, the production activity started to decline gradually (Fig. 6, yellow bars). As a result, the production yield dropped considerably by the 17th day, but even after that point negligible amount of sucrose was detected in the medium until the end of the experiment (Fig. 6). The beads remained stable during the 27 days of incubation and outgrowth from the beads remained inconsequential in the beginning of production but increased after 2 weeks of cultivation (up to OD_750_ 1.0 on the 13th day), and then continued to increase until the end of the experiment reaching the maximum OD_750_ 1.5 (Fig. 6). This can be attributed to the slow degradation and the gradual breach of the surface of the beads. Bead immobilization is a practical and effective method for semi-continuous cultivation since no energy-intensive centrifugation is needed and even on an industrial scale, the medium can be easily changed through a valve system. The cumulative maximum sucrose yield during the semi-continuous cultivation reached 3000 mg l^−1^ after 17 days (Fig. 6), which is almost three times higher compared to the amount obtained during 7 days of batch production. This clearly demonstrates that semi-continuous cultivation is a better alternative than batch cultivation to maximize the amount of harvested sucrose by prolonging the duration of the production period of *Synechocystis* S02 immobilized in Ca^2+^-alginate beads.

### Sucrose produced by *Synechocystis* drives biotransformation in *E. coli*

**Fig. 7:**
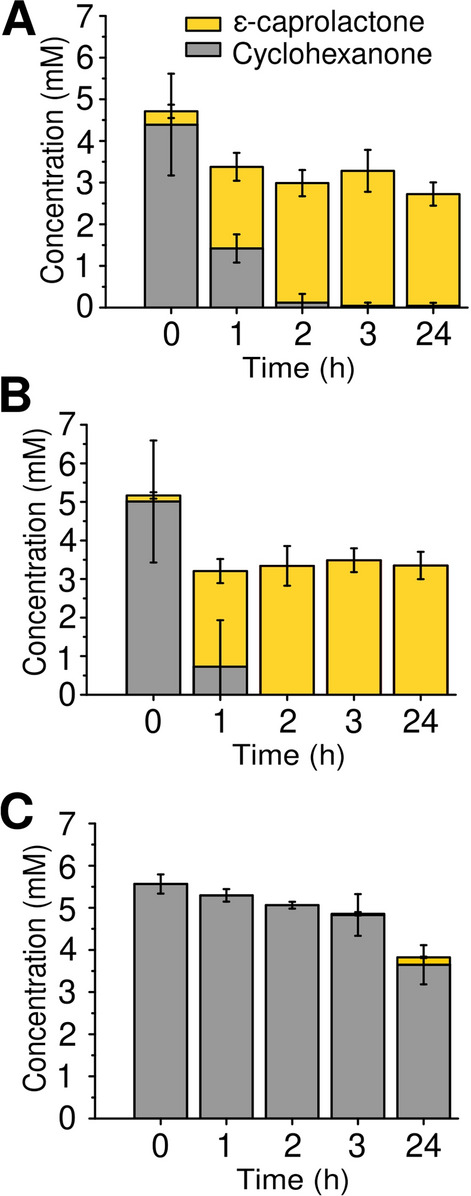
Progression of *E. coli* driven biotransformation of 5 mM cyclohexanone substrate to *ε*-caprolactone in different media. **A** BG11 + NaCl medium enriched with sucrose by alginate immobilized *Synechocystis* S02 cells; **B** M9 medium supplemented with 10 mM sucrose as positive control and **C** BG11 + NaCl medium without sucrose as negative control

The next step was to verify the capability of sucrose produced by immobilized *Synechocystis* S02 cells to sustain the biotransformation of cyclohexanone to *ε*-caprolactone in recombinant *E. coli*. For this purpose, we engineered *E. coli* WΔ*cscR* Inv, which is capable of effectively utilizing even low amounts of sucrose due to the deactivation of sucrose catabolism repressor gene (*cscR*) and heterologously expressed invertase enzyme with an N-terminal *pelB* leader sequence for export to the periplasm [32]. Expression of a heterologous Baeyer–Villiger monooxygenase from *Parvibaculum lavamentivorans* (BVMO_*Parvi*_) in the WΔ*cscR* Inv background enabled the biotransformation of cyclohexanone, exogenously added substrate, to *ε*-caprolactone by utilizing the sucrose produced by immobilized *Synechocystis* S02 (Fig. 7).

Sucrose was produced in BG11 + NaCl by alginate immobilized *Synechocystis* S02 cells over a 7-day period (1150 mg l^−1^). Then the medium was removed from the beads and used as culture medium for the biotransformation by the *E. coli* WΔ*cscR* Inv:Parvi strain. We monitored the biotransformation by the engineered *E*. *coli* cells to evaluate the potential of coupled production. Under these conditions, we observed full biotransformation of cyclohexanone to *ε*-caprolactone within three hours (Fig. 7A) with the average transformation rate of 0.9 mM h^−1^. M9 minimal medium used for the cultivation of *E*. *coli* was supplemented with 10 mM sucrose and used as a positive control for the biotransformation. The control reaction in M9 was faster, and proceeded to completion within 2 h, with the average rate of 2.3 mM h^−1^ (Fig. 7B). As a negative control fresh BG11 + NaCl medium without any additional sucrose was used to ascertain that the *E. coli* is not capable of performing the biotransformation reaction without sucrose, utilizing for example, energy stored during previous cultivation steps. Only minimal amount of cyclohexanone was converted to *ε*-caprolactone in the absence of sucrose over the 24-h time period (Fig. 7C). Since both the substrate (cyclohexanone) and the product (*ε*-caprolactone) are semi-volatile, the final concentration of the product can deviate from the initial substrate concentration. From these results it is evident that the *E. coli* WΔ*cscR* Inv:Parvi strain is capable of fast conversion of cyclohexanone to *ε*-caprolactone without the formation of side-product, cyclohexanol. The conversion rate is comparable to other *E. coli* strains harboring BVMOs when utilizing rich TB-medium [9]. This also confirms that an organic carbon source, in our case sucrose, is essential for the cyclohexanone biotransformation in *E*. *coli*, as expected based on the BVMO dependence on NADPH, which is generated via the glycolytic pentose phosphate pathway in this host. [33]. However, other components of the previously used TB-medium seem to be less important, while the high salt concentration is no hindrance for cells to perform the biotransformation under the used conditions. Altogether, the data unambiguously demonstrate that sucrose produced by *Synechocystis* S02 over a 1-week batch culture, is sufficient to drive the conversion of 5 mM cyclohexanone to *ε*-caprolactone by *E*. *coli* WΔ*cscR* Inv:Parvi without any downstream modification or manipulation of the medium.

Our findings support the general concept of utilizing carbohydrates synthesized from CO_2_ by engineered cyanobacteria as a source of energy for biotransformation catalyzed by heterotrophic microbes and lay the foundation for an alternative sustainable *ε*-caprolactone production platform. To effectively couple the photoautotrophic and heterotrophic production systems in actual co-cultures where the two process is simultaneously ongoing in one bioreactor, however, further optimization is needed. In contrast to the published co-cultures [8, 19–22] biotransformation offers ‘the substrate-in-product-out’ concept, where besides the sucrose produced by cyanobacteria also the external substrate has to be fed to the *E. coli* cells. In the published examples the compounds produced by the heterotrophs are products derived from the carbon fixed by the phototroph from CO_2_ and no compound is fed to the strain to be transformed. The CO_2_ that the phototroph converts to sucrose is either utilized for the synthesis of compounds or for the growth of the heterotroph [8, 19–22]. The issues that need to be addressed to establish a working co-culturing system are (*i*) the considerable difference between the rate of sucrose production and the biotransformation process. This challenge could be overcome by introducing the *E. coli* performing the biotransformation together with cyclohexanone at later stages of the sucrose production to ensure sufficient amounts of sucrose to sustain the biotransformation; (*ii*) the overall slow sucrose production rate. This could be addressed by further engineering the sucrose producer or by exploring different strains, such as the promising *Synechococcus* elongatus UTEX 2973 [18]; (*iii*) the effective collecting of the biotransformation product without interrupting the sucrose production and co-culturing as well as the (*iv*) ways to avoid losses of the substrate and the product of the biotransformation in a prolonged setup remain challenges, that need experimental trials to find ways to overcome them.

## Conclusions

Our present work demonstrates the viability of a coupled autotrophic–heterotrophic production system where the sucrose-producing *Synechocystis* S02 entrapped within Ca^2+^-alginate beads provides sucrose as organic carbon source to drive biotransformation of cyclohexanone to *ε*-caprolactone by the *E. coli* WΔ*cscR* Inv:Parvi strain. By employing the immobilization approach, we increased the specific sucrose productivity of *Synechocystis* S02 by 86% compared to suspension cultures. Furthermore, we demonstrated that the sucrose production in immobilized cultures can be prolonged for at least 17 days by applying a semi-continuous production system, in which the medium is refreshed periodically. Immobilization has a clear advantage over the suspension cultivation since it simplifies the change of medium in photobioreactors and, thus, makes the process less energy demanding. To emphasize the applicability of coupled culturing of sucrose-producing *Synechocystis* S02 strain and a heterotrophic microbe, sucrose was produced over 7 days in BG11 + NaCl medium. Subsequently, the medium was removed and used to drive biotransformation of cyclohexanone to *ε*-caprolactone by the *E. coli* WΔ*cscR* Inv:Parvi strain. The biotransformation went to near completion in three hours without formation of cyclohexanol side-product, showing that the sucrose in the medium sustains the reaction without the downstream processing. Our present work paves the way for further designing and optimization of a coupled autotroph–heterotroph production systems for *ε*-caprolactone production. Furthermore, the platform could be applicable for biosynthesis and biotransformation of other compounds using *E. coli*-based cell factories*.*

## Materials and methods

### Strains and culture conditions

*Synechocystis* sp. PCC 6803, strain S02 (*Synechocystis* S02) [14] was used for the production of sucrose in this study. The strain contains a heterologous plasmid for the expression of *cscB* sucrose permease gene from *E. coli* and *Synechocystis* SPS gene *sll0045* for overexpression and increased sucrose production. The plasmid contains spectinomycin (Sp) and chloramphenicol (Cm) resistance genes as selection markers. GGPS gene *sll1566* was inactivated by insertion of a kanamycin (Km) resistance cassette for preventing the synthesis of glucosylglycerol (Fig. 1).

Stock cultures were maintained in 100 ml Erlenmeyer flasks containing 25 ml BG11 medium supplemented with 20 mM HEPES, pH 7.5-NaOH (BG11) and antibiotics: 20 μg ml^−1^ Sp, 8 μg ml^−1^ Cm and 20 μg ml^−1^ km. The flasks were placed in the growth chamber supplemented with 1% CO_2_ in air and cultivated at 30 °C on an orbital shaker (100 rpm) under 25 μmol photons m^−2^ s^−1^ light.

Optical density (OD) of cultures was measured at 750 nm with the spectrophotometer (Genesys 10S UV–Vis, Thermos Scientific, US).

For the generation of WΔ*cscR* Inv:Parvi *E. coli* strain the pQE-30 expression plasmid harboring the synthetic Baeyer–Villiger monooxygenase gene from *Parvibaculum lavamentivorans* (BVMO_*Parv*i_), *cscA* invertase gene with an N-terminal *pelB* leader sequence (Inv) and, ampicillin resistance genes was purchased from GenScript. This was plasmid introduced to *E. coli* W Δ*cscR* strain, which was kindly provided by Prof. Claudia Vickers (University of Queensland, Australia). As the strain does not carry the gene for T7 polymerase, it was important to clone the BVMO_*Parvi*_:Inv cassette under the control of a different promoter than T7. Hence, IPTG inducible T5 bacteriophage promoter was used.

### Sucrose production in *Synechocystis* suspension cultures

Experimental cultures were grown in 250-ml Erlenmeyer flasks containing 100 ml BG11 medium without antibiotics in a growth chamber supplemented with 1% CO_2_. They were cultivated on an orbital shaker (100 rpm) at 30 °C under 200 μmol m^−2^ s^−1^ light. When the OD_750_ reached 1.0, the cells were pelleted and resuspended in 100 ml BG11 medium supplemented with 400 mM NaCl (BG11 + NaCl) to induce salt stress for sucrose accumulation. The cells were left overnight for acclimation and were pelleted again and resuspended in fresh BG11 + NaCl medium. Initial OD_750_ was set to 0.5. 1 mM IPTG was added to induce *cscB* expression for sucrose secretion.

### Sucrose production in *Synechocystis* immobilized in alginate beads

Immobilization of *Synechocystis* S02 cells in alginate beads was performed according to the work of Weiss and co-workers [8] with slight modifications. Suspension cultures were prepared as described above, but when the cultures reached OD_750_ 1.5 the cells were pelleted and resuspended in 1/24 of the original volume in 20 mM HEPES–NaOH pH 7.5. This suspension was added to 3% (wt/v) alginate solution (Sigma-Aldrich, US) in 1:12 ratio. Next, the cells were mixed homogeneously with a pipette tip and the mixture was loaded into a 10-ml syringe. The resulting cell density was doubled to the original suspension cultures (OD_750_ = 3). The final alginate concentration in the hydrogel matrix formulation was 2.75%. The alginate–cell mixture was dropped into a 50-ml centrifuge tube filled with 40 ml of 50 mM CaCl_2_ solution through a 25G (0.5*25 mm) hypodermic needle. The drops immediately congealed. The beads were left to solidify for 10 min after which the CaCl_2_ solution was discarded, and the beads were washed twice with MQ water. The diameter of the resulting beads was ~ 2.5 mm. Bead immobilized cells were placed in 75 cm^2^ filter capped culture flasks (NunC EasYFlask, Thermo Scientific, US) in BG11 + NaCl containing 1 mM IPTG. Each flask contained 10 ml beads and 40 ml medium.

### Biotransformation of cyclohexanone to *ε*-caprolactone with *E. coli* WΔ*cscR* Inv:Parvi

*E. coli* WΔ*cscR* Inv:Parvi strain was used to demonstrate the utilization of sucrose produced by alginate bead-immobilized *Synechocystis* cells. Overnight cultures were made in 5 ml LB medium supplemented with 100 µg ml^−1^ ampicillin and used to inoculate 200 ml TB medium. The cultures were grown at 37 °C with 200 rpm orbital shaking until they reached OD_600_ 2–3, then the expression of BVMO_*Parvi*_ was induced with 1 mM IPTG and cultures were incubated at 30 °C overnight. The next day the cells were collected and washed with 5 mM HEPES buffer pH 7.5. Cells were resuspended to OD_600_ 7–8 in 30 ml BG11 + NaCl medium enriched with sucrose by alginate bead-immobilized *Synechocystis* cells. This medium was collected directly from the beads and stored at −20 °C without centrifugation or other manipulation to assess the feasibility of co-cultures. M9 medium supplemented with 10 mM sucrose was used as positive control and BG11 was used as a negative control. Cultures were incubated in baffled Erlenmeyer flasks at 30 °C, 1% CO_2_ and 180 rpm orbital shaking with 5 mM cyclohexanone (Sigma-Aldrich, US). Samples were taken right after the addition of cyclohexanone and 1, 2, 3 and 24 h later. Samples were flash-frozen in liquid nitrogen and kept at −80 °C until extraction and analysis.

### Sucrose analysis

For short-term sucrose production, 0.5 ml samples were taken from the medium every day for 7 days. For long-term sucrose production, sampling was done every 3–4 days after which the medium was replaced with fresh BG11 + NaCl containing 1 mM IPTG. Samples were centrifuged and supernatants were stored at −80 °C until analysis. Sucrose concentration was measured using a commercial kit (sucrose/d-glucose Assay Kit, Megazyme, US).

### Determination of cyclohexanone and *ε*-caprolactone concentration in *E. coli* samples using gas chromatography (GC)

Extraction was done by ethyl acetate with 2 mM acetophenone (Sigma-Aldrich, US) as an internal standard. 300 µl sample with 0.06% (v/v) HCl was extracted twice with 150 µl ethyl acetate and shaken for 15 min with multi-tube vortex (Baxter, US). After centrifugation the ethyl acetate (Lab-Scan Analytical Sciences, Poland) phase was collected and dried with anhydrous MgSO_4_. The samples were centrifuged and measured with GC-2010 Pro gas chromatograph (Shimadzu, Japan) equipped with a HP-5MS 30 m × 0.25 mm (5%-Phenyl)-methylpolysiloxane column (19091S-133, Agilent) using nitrogen as carrier gas with splitless injection mode. Compounds were separated at 35 °C (hold 3 min), 200 °C (hold 3 min, 10 °C min^−1^) and 300 °C (hold 3 min, 25 °C min^−1^). Linear velocity was 11 cm sec^−1^. Calibration was done by using known amounts of cyclohexanone, *ε*-caprolactone and acetophenone (Sigma-Aldrich, US).

### Chlorophyll a measurement

Chlorophyll *a* (Chl) concentration of suspension cultures was measured by taking 1 ml sample and pelleting it by centrifugation. Next, the supernatant was removed, and the pellet was resuspended in 1 ml 90% methanol. The samples were incubated in the dark for 5 min and centrifuged. Absorbance of the supernatant was measured at 665 nm using UV-1800 spectrophotometer (Shimadzu, Japan). Chl concentration was calculated according to Lichtenthaler [34]. For Chl extraction from beads, 1 ml of beads were placed in a 15 ml centrifuge tube and 9 ml 100% methanol was added. The tubes were incubated at + 4 °C in darkness for 48 h. Measurements were done the same way as for the suspension cultures.

### Fluorescence measurements

PSII yield was measured using PAM 2000 Portable Chlorophyll Fluorometer (Walz Mess und Regeltechnik, Germany). Beads were placed on a Petri dish in one layer depth and the medium was removed. *Y(II)* was determined under a series of saturating light pulses of the same intensity, 3000 μmol photons m^−2^ s^−1^ for 0.8 s applied on top of 200 μmol photons m^−2^ s^−1^ actinic light.

### Membrane inlet mass spectrometry

Membrane inlet mass spectrometry (MIMS) was used to monitor carbon fixation and O_2_ evolution in suspension and bead-immobilized cultures in sucrose-exporting (+ NaCl) and non-exporting (−NaCl) conditions to investigate the effect of immobilization and sucrose secretion on the cells. Gas exchange was measured by Prima PRO membrane inlet mass spectrometer (Thermo Fisher, USA). Samples were loaded in a modified DW1 oxygen electrode chamber with a water jacket keeping the temperature of the samples at 30 °C. From bead-immobilized cells, 20 beads were loaded in the chamber and the medium used during the incubation and enriched by sucrose was added to a final volume of 2 ml. In case of suspension cultures, 2 ml was applied with a Chl concentration adjusted to 10 μg ml^−1^ with fresh BG11 or BG11 + NaCl medium, respectively. 1.5 mM of NaHCO_3_ was added to the samples and they were continuously stirred. A Teflon membrane was used to separate the sample chamber from the vacuum line. The presence of O_2_ (*m/z* 32 and 36) and CO_2_ (*m/z* 44) was monitored. ^18^O_2_ isotope was added in equal ratio to ^16^O_2_ to distinguish between respiratory oxygen uptake and O_2_ evolution resulting from water oxidation in PSII. Using this method, we were able to calculate total and net oxygen production. During the measurement, 5-min dark adaptation was followed by 5 min of illumination with halogenic light of 500 μmol m^−2^ s^−1^ intensity. Gas exchange rates were determined according to Beckmann et al. [35].

### Data treatment and statistics

Figures were created and fitted with Origin software (OriginLab Corporation, US). Analysis of significance was done by R (R Foundation for Statistical Computing, Austria). Student’s T-test was used for the statistical analysis of sucrose concentrations and independent sample T-test for gas exchange rates.

## Data Availability

The datasets used and/or analyzed during the current study are available from the corresponding author on reasonable request.
